# Climate Change Denial as Identity Defence: Understanding Resistance Beyond Ignorance

**DOI:** 10.1007/s00267-025-02353-5

**Published:** 2025-12-27

**Authors:** Ehsan Ebrahimi

**Affiliations:** 1 Faculty of Agriculture, Civil and Environmental Engineering, University of Rostock, Rostock, Germany; 2 Institute of Psychiatry, Psychology and Neuroscience, King’s College London, London, UK

**Keywords:** Belief, Climate change, Cognitive dissonance, Emotion, Identity

## Abstract

Climate denial is often misunderstood as ignorance, but evidence from neuroscience reveals it as identity protection. This perspective integrates insights from the neuroscience of belief, emotion, and decision-making into climate communication, arguing that resistance to climate action reflects *how* people process information, not *how much* they know. Scientific messages that conflict with people’s values or group identities often provoke reinterpretation or rejection of the facts. Climate change is also a uniquely abstract and psychologically distant threat and fear-based appeals often backfire unless paired with agency and clear solutions. More effective communication must be participatory, emotionally intelligent, and grounded in trust, aligning with audience values and reducing psychological threat. Approaches built on empathy, local context, and collaboration can make climate communication not only more persuasive but also transformative.

## Highlights

- Climate resistance reflects identity protection rather than a lack of knowledge.
- Neuroscience shows belief is emotionally rooted and socially shaped.
- Fear-based climate messages backfire unless paired with hope and agency.
- Climate threats are abstract, distant, and psychologically hard to grasp.
- Participatory, values-based communication builds trust and sustained engagement.

## Introduction

Those working in the fields of environmental science or agriculture have spent years collaborating with people on the frontlines of change, including farmers, policymakers, researchers, and local communities, to build more sustainable and resilient systems. But why do so many farmers who accept worsening droughts remain sceptical of climate change?

Despite decades of scientific evidence, climate communication strategies still rely heavily on rational-choice models, more data, better visuals, and clearer facts, yet behavioural resistance persists (Björnberg et al. 2017). This resistance has often been explained in terms of ignorance, misinformation, or scientific illiteracy. However, research in the social and communication sciences shows that climate scepticism and denial are deeply tied to identity, intergroup dynamics, and defence of the status quo (Sarathchandra et al. 2022; Hornsey 2020). Building on that foundation, insights from neuroscience and psychology reveal how such identity-based resistance operates in the brain through emotional processing, threat perception, and motivated reasoning (Toomey 2023; Davidson and Kecinski 2022; Manning et al. 2018). Resistance to climate information is therefore not a failure of knowledge, but a defence of emotion, identity and group cohesion.

This perspective integrates identity-based explanations of climate denial with neuroscience of belief and threat processing, and translates that into communication principles that reduce defensiveness. It calls for a shift away from fact-based persuasion toward strategies rooted in empathy, trust, and psychological safety. The goal is not to correct the public, but to reshape communication itself as a form of engagement with human identity. The limits of the ‘information deficit’ model and organised disinformation (where, in some populations, scientific consensus messaging can help by leveraging high trust in scientists) are increasingly acknowledged. Yet few climate communication approaches explicitly incorporate these neuropsychological insights regarding emotional processing and identity defence to explain why scientific information often fails to shift attitudes or behaviours.

In this text, climate denial refers not only to the rejection of scientific evidence but also to the denial of human causation or the need for collective action. Understanding denial in this sense moves the discussion beyond traditional behavioural models and highlights the need for communication approaches grounded in empathy, identity awareness, and trust-building.

## The Neuroscience of Belief

Climate communication often assumes that people form beliefs through logic and reason. Yet neuroscience tells a different story. Beliefs are not purely cognitive but are deeply intertwined with emotions and are embodied (Asp et al. 2012). Functional brain imaging shows that statements like ‘climate change is real’ or ‘organic farming is more sustainable’ activate brain regions involved in identity and self-reflection, including the default mode network and ventromedial prefrontal cortex (Wang et al. 2023).

**Fig. 1:**
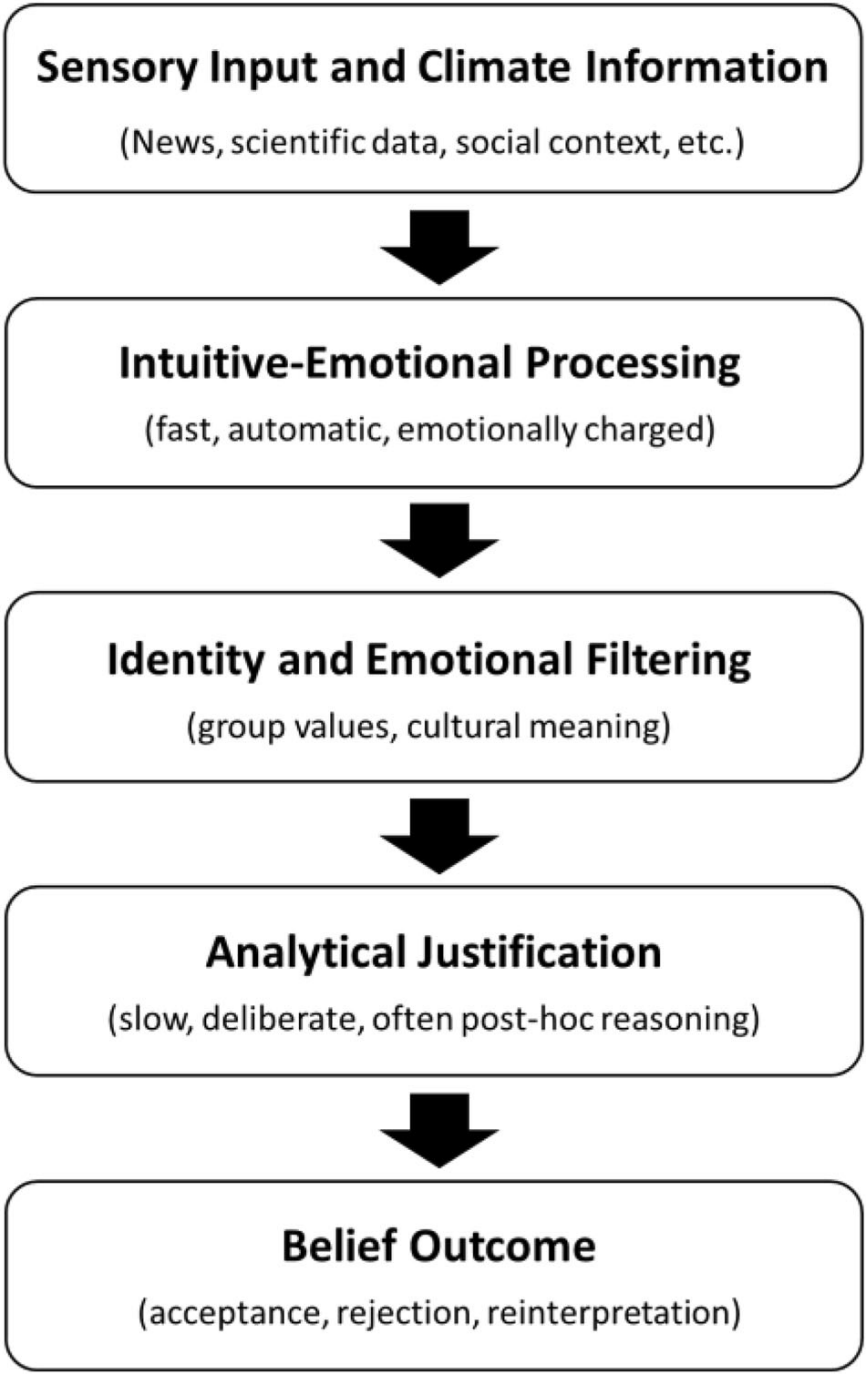
Belief formation and climate information filtering. Conceptual illustration by the author, informed by dual-process accounts of reasoning and research on identity-biased processing. Scientific messages are first processed through fast, intuitive, and emotion-driven pathways (System 1), which are shaped by personal identity, values, and group affiliation. This filtering strongly influences whether the slower, analytical reasoning system (System 2) is activated or biased

These findings suggest that beliefs feel like objective knowledge, not because they result from rational evaluation, but because of how the brain processes information. According to dual-process theory, beliefs are initially formed by fast, intuitive, and emotionally driven mechanisms. A second, slower system may later evaluate or justify them, but only when prompted (Fig. 1). When beliefs align with a person’s internal framework or social trust network, this rational review is often bypassed (Evans 2011; Frankish 2010). For instance, a farmer’s belief that climate change is exaggerated may stem from emotional group alignment rather than from a rational analysis of temperature data. This explains why some individuals remain indifferent to even well-substantiated warnings. When new information conflicts with prior intuitions or lived experiences, they may feel rejected, regardless of their empirical accuracy (Etana et al. 2021; Islam et al. 2013).

For environmental scientists, understanding these emotional roots is essential. Presenting data and scientific evidence alone will not shift behaviour if they clash with identity and values. If these underlying dimensions are overlooked, even the most accurate data may fail to influence people’s attitudes and behaviours. But understanding how beliefs are formed is only half the story. We must now examine how people protect those beliefs when they are challenged, especially when the threat is existential.

## Dissonance and Identity Defence

If beliefs are emotionally embedded and socially reinforced, what happens when they are challenged by new information? One of the brain’s common responses is cognitive dissonance, the uncomfortable tension that arises when incoming facts conflict with existing beliefs or emotions (Oswald and Bright 2021). Neuroscience shows that this dissonance activates areas of the brain associated with conflict detection (anterior cingulate cortex) (Tandetnik et al. 2021) and emotional distress (insula) (de Vries et al. 2015), creating a strong urge to resolve the tension.

To reduce this discomfort, people often engage in *motivated reasoning*, the tendency to interpret information in ways that protect *existing* beliefs. Freiling et al. (2023), in a large-scale COVID-19 misinformation study, found that when information clashes with identity or group affiliation, individuals selectively dismiss or reinterpret even clearly debunked facts. Comparable identity-protective dynamics are well documented in climate scepticism. Research shows that denial is sustained by deep motivational ‘attitude roots’ such as ideology, fear, and social-identity needs (Hornsey and Fielding 2017); that believers and sceptics form polarised socio-political identities reinforced by distrust in science (Bliuc et al. 2015; Bugden 2022); and that denial correlates with lower environmental identity and higher individualism across cultures (Nartova-Bochaver et al. 2022). Systematic reviews further highlight that effective counter-strategies depend on recognising the specific form of denial and tailoring communication to the audience identity (Mendy et al. 2024). These findings confirm that climate scepticism arises from identity-protective cognition rather than information shortage. For instance, some farmers reject long-term climate forecasts not because they doubt the data, but because acceptance would threaten cultural norms, economic models, or their professional self-concept (Islam et al. 2013). Therefore, what is being defended is not truth, but identity.

**Fig. 2:**
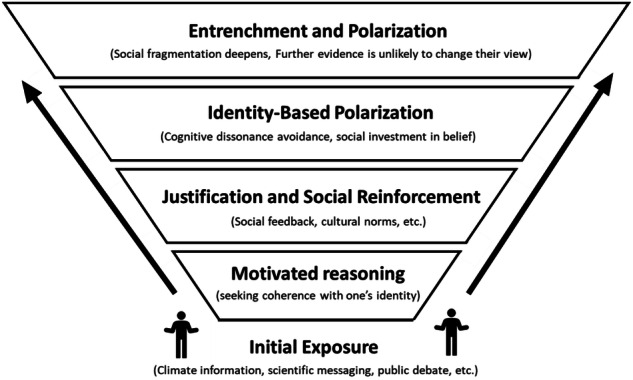
The layered psychological barriers to climate engagement. Conceptual illustration of the pyramid of choice model by the author, showing how climate information becomes filtered through multiple layers of cognitive defence, from initial discomfort and motivated reasoning to identity protection and group reinforcement

Directionally motivated reasoning, the process of aligning information with one’s worldview, explains why people hold fast to beliefs even when presented with contradictory evidence (Jost 2019). The ‘pyramid of choice’ model (Fig. 2) illustrates how small interpretive differences, reinforced by social media or partisan cues, can set individuals on divergent paths. Over time, this reinforcement deepens polarisation despite access to shared facts (Kovács et al. 2024; Tavris and Aronson 2007).

Understanding these mechanisms matters because resistance to climate science is not irrational or rooted in ignorance; it is an adaptive strategy to preserve coherence and control. For environmental scientists, this means that effective climate communication must do more than present facts; it must lower psychological threat, respect identity, and create space for dialogue and self-reflection.

## Climate Change is Uniquely Difficult

Climate change presents a unique communication challenge, not because the science is uncertain, but because the threat it represents is psychologically misaligned with how the human brain has evolved to perceive danger (Mobbs et al. 2007). Unlike immediate, personal, and visible threats, climate change is abstract, distant, gradual, and unevenly experienced, making it cognitively difficult to grasp and emotionally hard to prioritise (Spence et al. 2012).

Neuroscience helps explain this gap: the brain is attuned to local, near-term, and emotionally salient risks, not to statistical projections or invisible atmospheric shifts (Wang et al. 2019). Messages about sea-level rise, jet stream disruption, or temperature thresholds may engage analytical reasoning, but often fail to activate the emotional systems that motivate behaviour. This helps explain why even those who accept climate science intellectually may remain disengaged in practice.

The emotional disconnect is compounded by the frequent use of fear-based messaging. This appears on both sides of the climate debate: while science communication often emphasises catastrophic risks, disinformation campaigns likewise exploit fear to provoke resistance. The issue is not emotion itself, but how it is used. Fear can capture attention, but it rarely inspires action unless paired with clear solutions, relevance, and a sense of agency (Nabi and Myrick 2019). Without these, fear may provoke denial, inaction, or disengagement rather than motivation, a form of emotional self-protection.

Beyond emotion, climate communication is often engaged in identity and value conflicts. Many proposed solutions, from dietary changes to lifestyle shifts, are perceived not as scientific necessities but as ideological intrusions. Acceptance or rejection of climate science thus becomes less about evidence and more about belonging to a particular social identity (Bayes and Druckman 2021).

In such a context, simply delivering clearer facts is not enough. Climate change is also a crisis of perception, emotion, and identity, challenging not only what we know but who we are. Effective communication must therefore move beyond traditional frameworks toward empathy, cultural sensitivity, and participatory engagement, meeting people where they are cognitively, emotionally, and socially to build transformative understanding and trust.

## From Facts to Frameworks: Toward Emotionally Intelligent Climate Communication

Traditional models of climate communication that rely on logic and rational reasoning have shown limited success. Instead, effective communication must begin with how people actually process information, through personal values, narratives, and emotions. This does not mean abandoning scientific accuracy but aligning messages with how the brain engages with meaning. When communication connects with what people already care about, such as the land, health, livelihoods, or their children’s future, it becomes far more likely to resonate (Bayes and Druckman 2021). In ecological agriculture, for example, adaptation strategies gain traction when framed around soil health, food security, or farmer resilience, rather than abstract targets like greenhouse gas reduction (Islam et al. 2013). These human-centred approaches foster durable and emotionally grounded engagement.

A sense of agency and personal control is also critical. When people feel they can make a difference, psychological threat is reduced and motivation increases (Deci and Ryan 2000). In contrast, vague, unattainable, or distant solutions can evoke defensiveness.

Participatory strategies, such as community-based renewable energy programs (Ishola et al. 2024), collaborative climate projects (Samaddar et al. 2021), or storytelling rooted in lived experience (Lowery et al. 2020), help overcome these barriers. These initiatives succeed not because they simplify science, but because they integrate climate communication into local identities, values, and social meaning.

For environmental scientists and communicators, this means moving beyond one-way data transmission toward a co-creative model of meaning-making. This model is grounded in listening, empathy, and cultural awareness. Informed by neuroscience and psychology, such approaches are not just more effective, but essential or meeting the scale and complexity of the climate crisis.

## Conclusion

The gap between climate science and public engagement is not a failure of knowledge but of communication design. Belief is shaped by emotion, identity, and social meaning, so resistance to climate information often functions as a psychological defence rather than ignorance. To meet this challenge, communication must connect science with human experience, be participatory, emotionally intelligent, and grounded in trust. It should align with people’s values, offer agency, and reduce psychological threat.

Climate communication is a critical frontier of climate action: its success will determine our ability to engage not only minds, but also hearts and identities, rebuild trust, and catalyse the collective agency needed for a sustainable future.

## Data Availability

No datasets were generated or analysed during the current study.
